# Role of the *p*-Coumaroyl Moiety in the Antioxidant and Cytoprotective Effects of Flavonoid Glycosides: Comparison of Astragalin and Tiliroside

**DOI:** 10.3390/molecules22071165

**Published:** 2017-07-12

**Authors:** Xican Li, Yage Tian, Tingting Wang, Qiaoqi Lin, Xiaoyi Feng, Qian Jiang, Yamei Liu, Dongfeng Chen

**Affiliations:** 1 School of Chinese Herbal Medicine, Guangzhou University of Chinese Medicine, Guangzhou 510006, China; wtttx0304@163.com (T.W.); linqiaoqi@163.com (Q.L.); feng569901110@126.com (X.F.); jiangqiande920711@163.com (Q.J.); 2 Innovative Research & Development Laboratory of TCM, Guangzhou University of Chinese Medicine, Guangzhou 510006, China; 3 School of Basic Medical Science & Research Center of Basic Integrative Medicine, Guangzhou University of Chinese Medicine, Guangzhou 510006, China; rebecca-22222@163.com (Y.T.); gzhlym@gzucm.edu.cn (Y.L.)

**Keywords:** tiliroside, astragalin, *p*-coumaroyl, flavonoid glycoside, mesenchymal stem cells

## Abstract

The aim of this study was to explore the role of *p*-coumaroyl in the antioxidant and cytoprotective effects of flavonoid glycosides. The antioxidant effects of astragalin and tiliroside were compared using ferric ion reducing antioxidant power, DPPH• scavenging, ABTS•^+^ scavenging, •O_2_^–^ scavenging, and Fe^2+^-chelating assays. The results of these assays revealed that astragalin and tiliroside both exhibited dose-dependent activities; however, tiliroside exhibited lower IC_50_ values than astragalin. In the Fe^2+^-chelating assay, tiliroside gave a larger shoulder-peak at 510 nm than astragalin, and was also found to be darker in color. Both of these compounds were subsequently evaluated in a Fenton-induced mesenchymal stem cell (MSC) damaged assay, where tiliroside performed more effectively as a cytoprotective agent than astragalin. Tiliroside bearing a 6′′-*O*-*p*-coumaroyl moiety exhibits higher antioxidant and cytoprotective effects than astragalin. The 6′′-*O*-*p*-coumaroyl moiety of tiliroside not only enhances the possibility of electron-transfer and hydrogen-atom-transfer-based multi-pathways, but also enhances the likelihood of Fe-chelating. The *p*-coumaroylation of the 6"-OH position could therefore be regarded as a potential approach for improving the antioxidant and cytoprotective effects of flavonoid glycosides in MSC implantation therapy.

## 1. Introduction

Several new flavonoid glycosides bearing a *p*-coumaroyl (*p*-coumaryl) moiety, including kaempferol-3-*O*-[2-*O*-(trans-*p*-coumaroyl)-3-*O*-α-l-rhamnopyranosyl]-β-d-glucopyranoside [1], 8,3′,4′-trihydroxyflavone-7-*O*-(6′′-*O*-*p*-coumaroyl)-β-d-glucopyranoside [2], hirtacoumaroflavonoside (7-*O*-(*p*-coumaroyl)-5,7,4′-trihydroxy-6-(3,3-dimethyl allyl)-flavonol-3-*O*-β-d-glucopyranosyl- (2′′→1′′′)-*O*-*α*-l-rhamnopyranoside) [3], dihydrokaempferide-3-*O*-*p*-coumaroylhexoside-like flavanone, isorhamnetin-3-*O*-*p*-coumaroylglucoside, chrysoeriol-*p*-coumaroylhexoside-like flavone [4], delphinidin-3-(4′′′-*O*-trans-*p*-coumaroyl)-rutinoside-5-*O*-glucoside and petunidin-3-(4′′′-*O*-trans-*p*-coumaroyl)-rutinoside-5-*O*-glucoside [5], have recently been isolated from a wide range of medicinal and dietary plant materials. The pharmacological evaluation of several similar flavonoid glycosides bearing a *p*-coumaroyl moiety revealed that these compounds exhibit various beneficial effects [6,7]. For example, apigenin-7-*O*-β-d-(6′′-*p*-coumaroyl)-glucopyranoside has been reported to exhibit neuroprotective effects in an experimental ischemic stroke mode [8], whereas tiliroside has been reported to inhibit neuroinflammation [9] and acute inflammation [10]. Notably, all of these effects have been attributed to the antioxidant activity of these compounds [10,11,12]. However, the role of the *p*-coumaroyl moiety found in these flavonoid glycoside compounds in their antioxidant activity remains unknown, despite numerous studies towards the structure–activity relationships of flavonoids and flavonols [13,14,15]. In this study, we have selected astragalin and tiliroside as model compounds to evaluate the role of the coumaroyl moiety in the antioxidant activity of these compounds.

**Figure 1:**
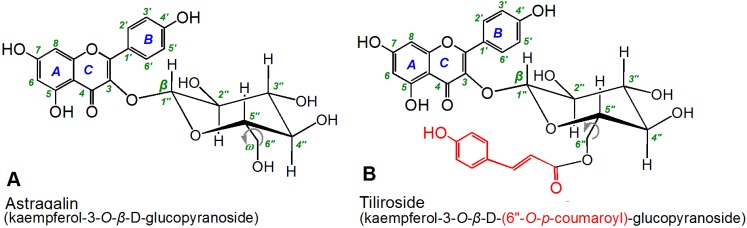
Structures of astragalin (**A**) and tiliroside (**B**).

Astragalin occurs naturally in *Zanthoxylum bungeanum* [16], *Flaveria bidentis (*L.*) Kuntze* [17], and *Morus alba* [18], whereas tiliroside can be found in *Tilia americana* L. (basswood) [19] and the Malvaceae family [20]. As shown in Figure 1, astragalin is actually kaempferol-3-*O*-β-d-glucopyranoside; whereas tiliroside is kaempferol-3-*O*-β-d-(6′′-*O*-*p*-coumaroyl)-glucopyranoside. The only difference between these two compounds is the *p*-coumaroyl moiety at the 6"-O position of tiliroside. A structure–activity relationship (SAR) analysis of these two compounds could therefore enhance our understanding of the role of *p*-coumaroyl moiety in the antioxidant activity of related flavonoid glycosides.

In this study, we investigated the SAR of these two compounds using several typical antioxidant models, including ferric ion reducing antioxidant power (FRAP), 1,1-diphenyl-2-picrylhydrazyl radical (DPPH•) scavenging, 2,2′-azino-bis(3-ethylbenzothiazoline-6-sulfonic acid radical cation (ABTS•^+^) scavenging, •O_2_^–^ radical anion-scavenging, and Fe-chelating UV spectroscopy assays. We also used mesenchymal stem cells (MSCs) to evaluate the cytoprotective effects of astragalin and tiliroside. MSCs could potentially be used in cell-based therapies for various diseases; however, a major problem in the clinical application of MSC-based therapies is the poor viability of transplanted MSCs at the site of the graft. This problem has been attributed to the harsh conditions associated with the microenvironment of the graft, including the increased production of reactive oxygen species (ROS). ROS can hinder cell adhesion and induce the detachment of cells, which can lead to anoikis signals in the implanted MSCs. The development of new strategies to regulate oxidative stress following the implantation of MSCs is therefore therapeutically attractive [21].

Coumaroylation can also occur in plant cell walls and coumaroylation status can be used as an indicator of the type of tissue in a plant [22,23]. With this in mind, the results of this study could also be used to develop a deeper understanding of the antioxidant defense system in plants.

Oxygen is widely distributed in the biosphere and can react to form various ROS, especially •OH and •O_2_^–^. Notably, ROS of this type can be found in nearly all of the animals and plants found on earth, where excessive ROS may bring about cellular oxidative damage. In plant cells, small molecule phytophenols act as antioxidants to eliminate excessive ROS [24]. Phytophenols, including the flavonoids typically found in Chinese herbal medicine, have also been used as effective natural antioxidants for the treatment and prevention of several human diseases.

## 2. Results and Discussion

**Figure 2:**
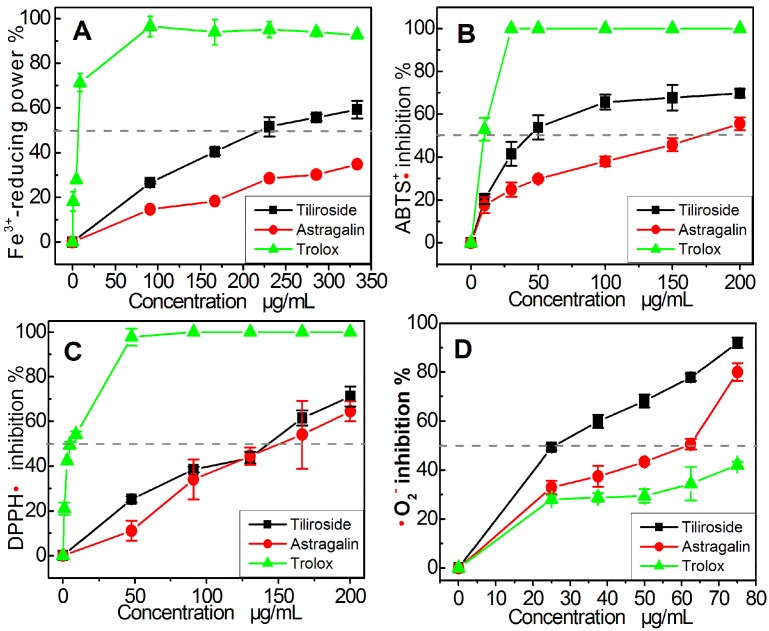
Dose-response curves of astragalin and tiliroside in various antioxidant assays: (**A**) FRAP assay; (**B**) ABTS scavenging assay; (**C**) DPPH•-scavenging assay; (**D**) •O_2_^–^-scavenging assay. Each value is expressed mean ± SD, *n* = 3. Trolox was used as the positive control.

Flavonoids are believed to scavenge ROS via multiple pathways, with electron transfer (ET) being regarded as one of the most common of these pathways [25,26,27]. This suggestion is also consistent with the fact that ROS are generated from oxygen through an ET process [24]. In this study, we used a FRAP assay to determine whether an ET pathway was responsible for the antioxidant activity of astragalin and tiliroside. As shown in Figure 2A, astragalin and tiliroside both gave good dose response curves for concentrations in the range of 0–348 μg/mL in the FRAP assay. These results suggested that these compounds operated via an ET pathway, because the FRAP assay was conducted under acidic conditions (pH 3.6), thereby inhibiting the deprotonation of the phenolic groups of the flavonoids. The IC_50_ values of astragalin and tiliroside were also found to be considerably different in the FRAP assay (Table 1). This result therefore implied that the *p*-coumaroyl moiety was enhancing the ET ability of tiliroside compared with astragalin.

A similar trend was also observed in the results of the ABTS scavenging assay, which indicated that the antioxidant activity mainly occurred via an ET reaction [28,29,30]. As shown in Figure 2B and Table 1, the trends in the dose–response curves of Trolox, astragalin, and tiliroside were similar to those observed in the FRAP assay. Furthermore, the relative antioxidant levels decreased in the order Trolox > tiliroside > astragalin. This further confirmed that at least one ET pathway was involved in the antioxidant activity of astragalin and tiliroside.

Astragalin and tiliroside were also analyzed using a DPPH scavenging assay. Previous reports have suggested that the DPPH-scavenging activities of different compounds mainly involve hydrogen atom transfer (HAT) pathways, leading to the formation of stable DPPH-H molecules [15]. However, several other minor pathways could also be involved in these scavenging processes, including ET, radical adduct formation (RAF), sequential electron proton transfer (SEPT), and proton coupled electron transfers (PCET) [31,32]. DPPH scavenging therefore involves a variety of different HAT-based pathways. As shown in Figure 2C and Table 1, astragalin and tiliroside both efficiently scavenged DPPH radicals; however, tiliroside showed higher DPPH radical scavenging ability than astragalin, indicating that its *p*-coumaroyl moiety enhanced the efficiency of the HAT-based pathways.

As a typical ROS, •O_2_^–^ can be scavenged through HAT, ET [33], proton transfer [34], and RAF [35] pathways. The dose-response curves in Figure 2D revealed that astragalin and tiliroside could both effectively scavenge •O_2_^–^ radicals. Similarly, the relative antioxidant levels of these compounds were of the order Trolox > tiliroside > astragalin (Figure 2D and Table 1). This result suggested that the *p*-coumaroyl moiety in tiliroside enhanced the possibility of multi-pathway-mediated •O_2_^–^ radical-scavenging processes.

It is well known that transit metal species (especially Fe^2+^) play an important role in the formation of ROS. For example, Fe^2+^ can catalyze the Fenton reaction to yield •OH Radicals (1) [36].

Fe^2+^ + H_2_O_2_ → Fe^3+^+ •OH + OH^–^ (1)

**Figure 3:**
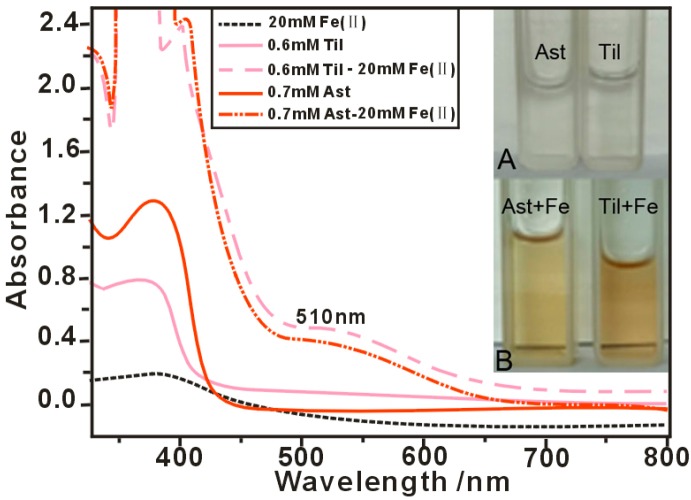
UV spectra of astragalin and tiliroside (**A**); and the physical appearances of the astragalin-Fe and tiliroside-Fe complexes (**B**).

The introduction of Fe^2+^-chelating groups could therefore be used as an efficient strategy to reduce the formation of ROS and enhance the antioxidant activity of flavonoids [36]. Furthermore, Fe^2+^-chelating has been developed as a therapeutic approach for many diseases related to ROS [37]. The results of the Fe^2+^-chelating assay conducted in the current study revealed that astragalin and tiliroside both gave a shoulder peak around 510 nm and became much darker in color when they were mixed with Fe^2+^(Figure 3). Furthermore, the UV absorbance spectra of these solutions shifted to a longer wavelength. This implied that the Fe^2+^-chelating ability of these compounds was acting as an indirect pathway to scavenge ROS. However, these results also suggested that tiliroside possessed higher Fe^2+^-chelating activity. This difference was attributed to the *p*-coumaroyl moiety of tiliroside.

**Figure 4:**
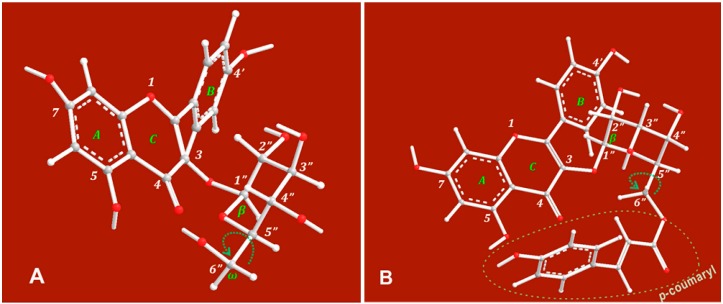
Ball-and-stick models based on preferential conformation of astragalin (**A**) and tiliroside (**B**).

**Figure 5:**
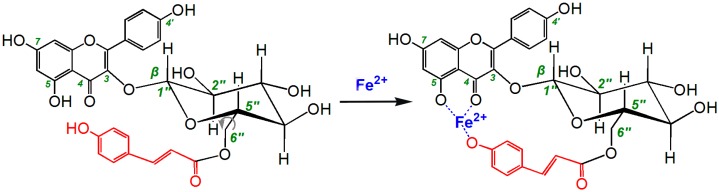
Proposed reaction of tiliroside chelating Fe^2+^.

As shown in the ball-and-stick models in Figure 4, the 6′′-O preferentially sat in an equatorial position (e bond) (Figure 4). This orientation placed the 6′′-O in close proximity to the flavone moiety (especially the A and C rings), allowing the *p*-coumaroyl moiety at 6′′-O to participate in binding interactions with 4-position and with 5-position via the free rotation of the σ bond between the 5′′- and 6′′-positions (Figure 5).

This would allow the conjugated *p*-coumaroyl moiety to reinforce the pentacyclic Fe^2+^-chelating around the 4- and 5-positions. These structural considerations therefore explain why the peaks in the UV spectrum of tiliroside were much more intense than those of astragalin and why it formed a much darker solution. It must be emphasized that the 6′′-*O*-*p*-coumaroyl moiety would not be able to access the 4′-OH and 7-OH positions to form a complex with Fe^2+^ because this would not allow for the formation of a pentacycle or hexacycle.

**Figure 6:**
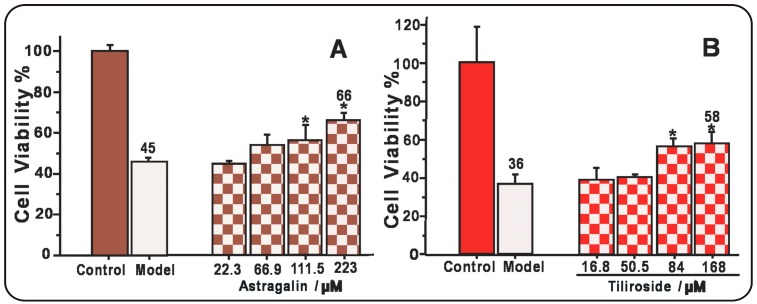
Protective effects of astragalin (**A**) and tiliroside (**B**) against the •OH-induced damage of MSCs using an MTT assay. The •OH radicals were generated by Fenton reagent (FeCl_2_ plus H_2_O_2_). These data represent the mean ± SD (*n* = 5). * *p* < 0.05 vs model.

Finally, we used an MSC-based model to evaluate the cytoprotective effects of astragalin and tiliroside. According to this model, the MSCs were initially oxidatively damaged using a Fenton reaction (i.e., FeCl_2_ plus H_2_O_2_) to generate •OH radicals. The results revealed that astragalin and tiliroside both protected the MSCs from •OH radical-induced damage. These results therefore suggested that astragalin and tiliroside exhibited cytoprotective effects towards MSCs. However, tiliroside was slightly more effective than astragalin, since 168.0 μM tiliroside could increase 20 percent points (38.8→58.0%) cell viability and such increase (46.1→68.5%) required 223.0 μM astragalin (Figure 6). Previous reports have shown that tiliroside can inhibit the oxidation of human low density lipoprotein [38] and inflammation in lipopolysaccharide-activated RAW 264.7 macrophages [39], both of which can be rationalized by the results of the current study. However, it is noteworthy that the structure of tiliroside was incorrectly presented in a previous report [39]. Our findings could also be used to develop a deeper understanding of the role of the *p*-coumaroyl moiety in plant physiology [22,23].

## 3. Materials and Methods

### 3.1. Animals and Chemicals

Sprague-Dawley (SD) rats of four weeks of age were obtained from the Animal Center at the Guangzhou University of Chinese Medicine, China. Tiliroside (C_30_H_26_O_13_, M.W. 594.52, CAS number: 20316-62-5, 98%) and astragalin (C_21_H_20_O_11_, M.W. 448.38, CAS number: 480-10-4, 98%) were obtained from Sichuan Weikeqi Biological Technology Co., Ltd (Chengdu, China). Pyrogallol, 2,4,6-tripyridyl triazine (TPTZ), and (±)-6-hydroxyl-2,5,7,8-tetramethlychromane-2-carboxylic acid (Trolox) were obtained from Sigma-Aldrich (Shanghai, China). 1,1-Diphenyl-2-picrylhydrazyl radical (DPPH•) was obtained from Aladdin Chemical, Ltd. (Shanghai, China). Tris-hydroxymethyl amino methane (Tris) was obtained from Dinggguo Biotechnology, Ltd. (Beijing, China). 2,2′-Azino-bis(3-ethylbenzothiazoline -6-sulfonic acid diammonium salt [(NH_4_)_2_ABTS] were obtained from Amresco Inc. (Solon, OH, USA). Dulbecco’s modified Eagle’s medium (DMEM) and fetal bovine serum (FBS) were purchased from Gibco (Grand Island, NY, USA). CD44 and 3-(4,5-dimethyl-2-thiazoyl)- 2,5-diphenyl-2-*H*-tetrazolium bromide (MTT) was from Duchefa were purchased from Boster, Ltd. (Wuhan, China). FeCl_2_·4H_2_O, K_2_S_2_O_8_, FeCl_3_·6H_2_O, Na_2_EDTA, hydrochloric acid, and all of the other reagents were purchased as the analytical grade from Guangdong Guanghua Chemical Plants Co., Ltd. (Shantou, Country).

### 3.2. Ferric Ion Reducing Antioxidant Power (FRAP) Assay

The FRAP assay was based on the method of Benzie and Strain [40]. In brief, the assay was performed in pH 3.6 buffer. Briefly, according to ratio of 1:1:10, the FRAP reagent was freshly prepared by mixing together 10 mM TPTZ and 20 mM FeCl_3_ in 0.25 M HOAc-NaOAc buffer (pH 3.6). The test sample (x = 10–50 μL, 1 mg/mL) was added to 100 μL of FRAP reagent. The absorbance was read at 593 nm after 2 h of incubation at 37 °C against a blank consisting of acetate buffer. The relative reducing power of the sample compared with the maximum absorbance was calculated using the following formula.

(2) $$\text{Relative reducing power}\;\%=\frac{A-A_{\min}}{A_{\max}-A_{\min}}\times 100\%$$

where, A_max_ is the maximum absorbance in this experiment, A_min_ is the minimum absorbance in this experiment, and A is the absorbance of sample.

### 3.3. ABTS ·^+^ Radical Scavenging Assay

ABTS ·^+^ scavenging activity was evaluated by the method [41]. The ABTS ·^+^ was produced by mixing 200 μL ABTS diammonium salt (7.4 mM) with 200 μL K_2_S_2_O_8_ (2.6 mM). After incubation in the dark for 12 h, the mixture was diluted with methanol (about 1:50) so that its absorbance at 734 nm was 0.3 ± 0.02. Then, the diluted ABTS ·^+^ solution (80 μL) was brought to 20 μL astragalin and tiliroside methanolic solution at various concentrations, thoroughly mixed. After the reaction mixture stood for 6 min, the absorbance at 734 nm was read on a spectrophotometer. The ABTS ·^+^-scavenging activity of each solution was calculated as percent inhibition, according to the equation

(3) $$\mathrm{Inhibition}\%=\frac{A_{0}-A}{A_{0}}100\%$$

where A_0_ indicates the absorbance of the blank and A indicates the absorbance of the sample.

### 3.4. DPPH• Radical Scavenging Assay

Scavenging activity on DPPH• radicals was assessed according to the method reported by Li [42]. Briefly, 5–25 μL of the sample methanolic solution (at least five different concentrations were prepared) was mixed with 100 μL DPPH• solution (prepared daily) in a 96-well plates. The mixture was shaken vigorously and left to stand for 30 min in the dark, and the absorbance was then measured at 519 nm by ELIASA (Thermo, Shanghai, China).The percentage inhibition was calculated by the formula above.

### 3.5. •O_2_^−^ Radical Scavenging Assay

The superoxide anion (•O_2_^−^)-scavenging activity was determined using a method previously developed in our laboratory [43]. Briefly, a 50–150 μL sample solution (0.5 mg/mL) was added to Tris-HCl buffer (0.05 M, pH 7.4) containing Na_2_EDTA (1 mM) and the total volume was adjusted to 990 μL using buffer. Ten microliters of pyrogallol solution (60 mM in 1 mM HCl) was added to the sample, and the resulting mixture was vigorously agitated before being analyzed at 325 nm every 30 s for 5 min by a UV spectrophotometer (Unico 2100, Shanghai, China). The •O_2_^−^ radical-scavenging ability was calculated as

(4) $$\mathrm{Inhibition}\;\%\;=\frac{\left(\frac{\Delta A_{325\mathrm{nm},\mathrm{control}}}{T}\right)-\left(\frac{\Delta A_{325\mathrm{nm},\mathrm{sample}}}{T}\right)}{{\left(\frac{\Delta A_{325m,\mathrm{control}}}{T}\right)}_{}}\;\times \;100\;\%$$

where ΔA_325 nm, control_ is the increase in the A_325 nm_ value of the mixture without the sample, ΔA_325 nm, sample_ is the increase in the A_325 nm_ value of the mixture with the sample and T is the time required for the determination (5 min in this case). 

### 3.6. Ultraviolet (UV) Spectra Determination of Fe^2+^-Chelating

The Fe-binding effects of astragalin and tiliroside were evaluated by UV spectroscopy. In these experiments, the Fe-binding reactions between astragalin and tiliroside were monitored based on their UV spectra. Briefly, 250 μL methanolic solution of tiliroside (2 mg/mL) or astragalin (2 mg/mL) was added to 1.5 mL of an aqueous solution of FeCl_2_·4H_2_O (5 mg/mL) and mixed vigorously. The resulting mixture was then incubated at 37 °C for 10 min. The product mixtures were photographed using a camera (Samsung GALAXY A7, Huizhou, China). The supernatant of each mixture was collected and analyzed on a UV–Vis spectrophotometer (Jinhua 754 PC, Shanghai, China).

### 3.7. Protective Effect Towards the •OH-Induced Damage of MSCs (MTT Assay)

The MSCs were cultured according to a previously reported method [44] and then oxidatively damaged by Fenton reagents, which were used to generate •OH radicals; the most harmful form of ROS. Briefly, bone marrow samples were obtained from the femurs and tibias of rats, and the resulting samples were diluted with DMEM (LG: low glucose) containing 10% FBS. The MSCs were obtained by gradient centrifugation at 900 g/min for 30 min on a 1.073 g/mL Percoll system. The cells were then detached by treatment with 0.25% trypsin and passaged into culture flasks at a density of 1 × 10^4^ cells/cm^2^. The homogeneity of the MSCs was evaluated at the third passage based on their CD44 expression by flow cytometry. These cells were then used for the following experiments.

The cultured MSCs were seeded into 96-well plates (4 × 10^3^ cells/well). After adherence for 24 h, the cells were divided into three groups, including control, model, and sample groups. The MSCs in the control group were incubated for 24 h in DMEM. The MSCs in the model group were injured for 5 min using FeCl_2_·4H_2_O (100 μM) followed by H_2_O_2_ (50 μM). The resulting mixture of FeCl_2_·4H_2_O and H_2_O_2_ was removed and the MSCs were incubated for 24 h in DMEM. The MSCs in the sample groups were injured and incubated for 24 h in DMEM in the presence of various concentrations of astragalin and tiliroside. After being incubated, the cells were treated with 20 μL of MTT (5 mg/mL in PBS), and the resulting mixtures were incubated for 4 h. The culture medium was subsequently discarded and replaced with 150 μL of DMSO. The absorbance of each well was then measured at 490 nm using a Bio-Kinetics plate reader (PE-1420; Bio-Kinetics Corporation, Sioux Center, IA, USA). The serum medium was used for the control group and each sample test was repeated in five independent wells.

### 3.8. Statistical Analysis 

The results were reported as the mean ± SD of three independent measurements, the IC_50_ values were calculated from dose−response curves and independent-samples T tests were performed to compare the different groups. A P value of less than 0.05 was considered statistically significant. Statistical analyses were performed using the SPSS software 17.0 (SPSS Inc., Chicago, IL, USA) for windows. All of the linear regression analyses described in this paper were processed using version 6.0 of the Origin professional software.

## 4. Conclusions

Taken together, the results of the current study have shown that tiliroside bearing a 6′′-*O*-*p*-coumaroyl moiety exhibits much greater antioxidant and cytoprotective activities than astragalin. The 6′′-*O*-*p*-coumaroyl moiety therefore not only enhanced the ET and HAT-based pathways available to this compound, but also enhanced its Fe^2+^-chelating ability. The *p*-coumaroylation of the 6′′-OH moiety of flavonoid glycosides therefore represents a useful strategy for the development of novel antioxidant and cytoprotective agents for MSC implantation therapy.

## Figures and Tables

**Table 1:** The IC_50_ values of astragalin and tiliroside in various assays.

Assay	Tiliroside μg/mL (μM)	Atragalin μg/mL (μM)	Trolox μg/mL (μM)
Fe^3+^-reducing	246.8 ± 19.3 ^b^ (550.5 ± 42.9) ^b^	465.8 ± 16.3 ^c^ (1038.9 ± 36.4) ^c^	6.8 ± 0.4 ^a^ (26.3 ± 1.8) ^a^
ABTS•^+^ scavenging	57.6 ± 8.9 ^b^ (96.8 ± 14.9) ^b^	170.7 ± 16.0 ^c^ (332.4 ± 11.1) ^c^	8.6 ± 2.5 ^a^ (34.3 ± 10.0) ^a^
DPPH• scavenging	138.0 ± 5.6 ^b^ (232.2 ± 9.4) ^b^	144.1 ± 25.1 ^c^ (321.3 ± 55.8) ^c^	6.8 ± 0.9 ^a^ (27.4 ± 3.5) ^a^
•O_2_^−^ scavenging	26.6 ± 2.3 ^a^ (44.8 ± 3.9) ^a^	45.7 ± 3.6 ^b^ (102.0 ± 8.0) ^b^	109.2 ± 8.9 ^c^ (436.3 ± 35.9) ^c^

**Note:** Each IC_50_ value was calculated from dose−response curves in Figure 2. The mass units of the IC_50_ values (μg/mL) were converted to molar unit, and the resulting values are shown in parentheses. The linear regression was analyzed using version 6.0 of the Origin professional software. Each experiment was performed in triplicate, and the IC_50_ values were presented as the mean ± SD (standard deviation, *n* = 3). Means values (μM) with different superscripts in the same row were significantly different (*p* < 0.05). Trolox was used as the positive control.

## Abbreviations

The following abbreviations are used in this manuscript:

DMEM	Dulbecco’s modified Eagle’s medium
DMSO	dimethyl sulfoxide
DPPH	1,1-diphenyl-2-picrylhydrazyl radical
ET	electron transfer
FBS	fetal bovine serum
FRAP	ferric ion reducing power assay
MSCs	mesenchymal stem cells
MTT	3-(4,5-dimethyl-2-thiazoyl)-2,5-diphenyl-2-*H*-tetrazolium bromide
RAF	radical adduct formation
ROS	reactive oxygen species
SAR	structure–activity relationship
SD	standard deviation
SPSS	statistical product and service solutions
TPTZ	2,4,6-tripyridyl triazine
Tris	tris-hydroxymethyl amino methane
Trolox	(±)-6-hydroxyl-2,5,7,8-tetramethlychroman-2-carboxylic acid
